# Prevalence and Clinical Significance of Hepatitis B Basal Core Promoter and Precore Gene Mutations in Southern Iranian Patients

**Published:** 2010-12-01

**Authors:** Seyed Alireza Taghavi, Manouchehr Tabibi, Ahad Eshraghian, Hossein Keyvani, Hamed Eshraghian

**Affiliations:** 1 Gastroenterohepatology Research Center, Shiraz University of Medical Science, Shiraz, IR Iran; 2 Internal medicine department, Shiraz University of medical science, Shiraz, IR Iran; 3 Microbiology department, Iran University of medical science, Tehran, IR Iran

**Keywords:** Basal core mutation, Precore mutation, Hepatitis B Virus, Iran

## Abstract

**Background and Aims:**

To investigate the prevalence and pattern of PC and BCP mutations and their clinical significance in patients with genotype D chronic hepatitis B infection in the Fars province of southern Iran.

**Materials and Methods:**

From January 2007 to March 2008, we evaluated 44 patients with chronic hepatitis B infection who were referred to our hepatology clinics affiliated with the Shiraz University of Medical Science. All Patients were HBeAg Negative and HBeAb positive. Basal core promoter and precore mutations in these patients were evaluated with clinical phenotype and laboratory tests.

**Results:**

The mean age of the patients was 37.21 ± 10.54 years. Twenty-seven patients (61.4%) had no mutations, whereas 17 patients (38.6%) had mutations in the precore or basal core promoter regions or both. The mean serum ALT level in mutation-free patients was 59.74 ± 55.86 IUL, whereas patients with PC and BCP mutations had a mean serum ALT level of 71.35 ± 59.49 IUL. The mean serum AST level in patients with mutations was higher than for patients without mutations (59.53 ± 41.35 IUL vs. 40.65 ± 25.21 IUL, respectively). There was no statistically significant difference between the mutation and mutation-free groups in terms of age, sex, and liver enzyme levels (P > 0.05). Fourteen of the 44 patients (31.8%) had mutations in the precore region (G 1896A). 17 patients (38.6%) had mutations in basal core promoter region.

**Conclusion:**

This study revealed a high prevalence of precore and basal core promoter mutations in southern Iran. Although no statistically significant difference was noted in liver enzymes, patients with mutations had higher liver enzymes in comparison with mutation-free patients.

## Introduction

Hepatitis B Virus (HBV) is a well-known agent of acute and chronic liver disease, and persistent HBV infection is closely associated with the development of cirrhosis and hepatocellular carcinoma. Around 400 million people worldwide carry the virus, and more than 250 million reside in Asia [1]. Based on the divergence of nucleotide sequences exceeding 8% in the entire genome or 4% in the S gene, HBV has been classified into eight genotypes designated by capital letters A through H [2]. These genotypes show variation in their geographic distribution [3]. Furthermore, some studies have explained the correlation between these genotypes, the clinical course of the disease, and responses to treatment [4].In the natural course of chronic HBV infection, the loss of HBeAg expression and the appearance of antibodies directed against it (Anti-HBe) are usually accompanied by a cessation of viral replication. However such a serological profile may also be seen in individuals who harbor precore (PC) and basal core promoter (BCP) mutants in which replicative infection continues. Mutations in the BCP of the HBV (T1762/A1764) and PC region (A1896) were previously reported to be linked with HBe antigen seroconversion (SC) and viral replication. They were often found in the patients with advanced liver disease and hepatocellular carcinoma. Considering the important effect of PC and BCP mutations on the course of the disease and especially on the efficacy of different types of treatment and the similarity of the serological profile to wild-type inactive HBV (negative HBe Ag, positive Anti-HBe Ab), accurate and reliable data about the prevalence and types of PC and BCP mutations and their impacts on the course of chronic hepatitis in Iranian patients are greatly needed.The present study aimed to determine the prevalence and pattern of PC and BCP mutations and their clinical significance in patients with genotype D chronic HBV infection in the Fars province of southern Iran.

## Materials and Methods

### Patients

From January 2007 to March 2008, 44 HBsAg-positive patients were referred to our outpatient liver clinics at the Shiraz University of Medical Science in the Fars province of Iran. All of the patients had chronic hepatitis B infection (defined as more than 6 months of infection) and all were HBeAg negative and HBeAb positive. None of the patients had associated infections (such as HCV, HDV, and HIV) or concomitant liver diseases including autoimmune hepatitis, Wilson's disease, primary biliary cirrhosis, alcoholic liver disease, and nonalcoholic fatty liver disease. None of the patients had received Lamivudin or interferon as a treatment for hepatitis B.

### Laboratory tests

During the initial visit, measurements of serum aspartate aminotransferase (AST) and alanine aminotransferase (ALT) levels were obtained from all 44 patients. HBeAg and anti-HBe were measured with commercially available kits (AxSYM,Abbott Laboratories, North Chicago, IL, USA) with a micro-ELISA method. HBV DNA was extracted from 200 μl of serum using a QIAamp DNA Blood Mini Kit (Qiagen GmbH, Germany) and amplified by an in-house PCR assay with a detection limit of 1000 geq/ml.

### Determination of PC and BCP mutations

The PC and BCP mutations were determined using a line probe assay (INN0-LIPA HBV PC), developed by INNOGENETICS (Ghent, Belgium). The PC and BCP regions were amplified by nested PCR with biotinylated primers provided by the manufacturer. Five microliters of the first-and second-round PCR products were analyzed by electrophoresis in a 2% agarose gel stained with ethidium bromide and visualized with an ultraviolet transilluminator. For samples with detectable HBV DNA after nested PCR, 10 microliters of amplified products were applied to the strips, which were precoated with specific oligonucleotide probes for wild-type HBV (A1762/G1764) and frequently occurring mutations in the core-promoter region (A1762/A1764, A1762/T1764 and T1762/A1764), and probes for mutant PC codon 28 (TAG). Following reverse hybridization of the bitinylated PCR fragments in the LiPA strips, streptavidin-alkaline phosphatase incubation and color development were used to identify reactive probes.

### LiPA

Equal volumes (10 microliters) of the biotinylated PCR fragment and the denaturation solution were mixed in test troughs for 5 min, after which 2 ml of the prewarmed hybridization solution was added, followed by the addition of one strip per trough. Hybridization occurred for 1 hr at 50±5º C in a closed water bath with back-and-forth shaking. The strips were rinsed twice with 2 ml of rinse solution. Strips were incubated on a rotating platform with alkaline phosphatase- labeled streptavidin conjugate for 30 min at 20 to 25 ºC. Strips were then washed twice with rinse solution, and color development was initiated with the addition of 5-bromo-4-coro-3-indolyphosphate (BCIP) and stopped by aspirating the substrate buffer and adding distilled water. Immediately after dying, the strips were interpreted.

### Ethics and consent

This study was approved by the Ethics Committee of the Shiraz University of Medical Science, where the work was undertaken. The study also conforms to the provisions of the Declaration of Helsinki (as revised in Edinburgh 2000).

### Statistical analysis

Results are expressed as mean ± SD. Data were analyzed by independent sample t-tests, two-tailed Fisher's exact and Chi Square tests when appropriate using SPSS software (version 12.0, SPSS Japan Inc., Tokyo). P values of < 0.05 were considered statistically significant.

## Results

All of the patients had genotype D HBV infection. Of the 44 patients, 11 individuals (25%) were female and 33 individuals (75%) were male. The mean age of patients was 37.21 ± 10.54 years. Twenty-seven patients (61.4%) had no mutations, whereas 17 patients (38.6%) had mutations in the PC and BCP regions. The mean age of patients with mutations was 38.35 ± 10.65 years, whereas the mean age was 36.07 ± 10.43 in mutation-free patients. The mean serum ALT level in mutation-free patients was 59.74 ± 55.86 IUL, whereas patients with PC and BCP mutations had serum ALT level of 71.35 ± 59.49 IUL. The mean serum AST level in patients with mutations was higher than the mean level in patients without mutations (59.53 ± 41.35 IUL vs. 40.65 ± 25.21 IUL, respectively). There was no statistically significant difference between the two groups in terms of age, sex, and liver enzyme levels (P > 0.05; Table 1).

**Table 1:** Age, sex, and liver enzymes in patients with and without PC and BCP mutations

**Mutation**			
	**With PC b and BCP c mutations**	**Without PC and BCP mutations**	**p-value**
Age	38.35 ± 10.65	36.07± 10.43	0.9
Sex (M/F)	22/5	11/6	0.289
ALT^a^	71.35 ± 59.49	59.74 ± 55.86	0.516
AST a	59.53 ± 41.35	40.65 ± 25.21	0.061

^a^ AST:Aspartate aminotransferase, ALT:Alanine aminotransferase

^b^ PC:Precore

^c^ BCP:Basal core promoter

Fourteen of the 44 patients (31.8%) had mutations at the PC region. All of these 14 patients had mutations at the same position (G 1896A). Seventeen patients (38.6%) had mutations in the BCP region, and among them, 14 patients had simultaneous mutation in the PC region and 3 of them had mutations only in the BCP region.The patients were divided in to five classes according to the patterns of their mutations. Class 0 included 27 patients (61.4%) with no mutations. Class 1 included 3 patients (6.8%) with a PC mutation at (G1896A) and a BCP mutation at (A1762/A1764). Class 2 included 8 (18.2%) patients with a PC mutation at (G1896A) and a BCP mutation at (T1762/A1764). Three patients (6.8%) who had a BCP mutation at (T1762/A1764) but no mutation in the PC region were categorized into Class 3. Finally Class 4 consisted of 3 patients (6.8%) with a PC mutation at (G1896A) and a BCP mutation at (T1762/G1764; Table 2). The most common BCP mutation in the 17 patients with any kind of BCP mutation was a dual T1762-A1764 mutation (25%).

**Table 2:** Age, sex, number and percent of patients in five classes

**Groups**	**0^a^**	**1^b^**	**2^c^**	**3^d^**	**4^e^**
Age	36.07 ± 10.43	34.67 ± 5.69	44.25 ±12.17	31.67 ±9.07	33 ± 2
Sex (M/F)	22/5	2/1	5/3	2.1	2/1
Number	27	3	8	3	3
Percent	61.4	6.8	18.2	6.8	6.8
ALT	59.74 ± 55.86	114 ± 117.85	49.63 ± 20.18	83.33 ± 76.00	74.67± 48.76
AST	40.56 ±25.21	100.33 ±73.21	48.13 ±30.22	46.67 ±23.03	62 ±36.29

^a^ 0=No mutation

^b^ 1=PC mutation (G1896A) + BCP mutation (A1762/A1764)

^c^ 2=PC mutation (G1896A) + BCP mutation (T1762/A1764)

^d^ 3=BCP mutation (T1762/A1764)

^e^ 4=PC mutation (G1896A) + BCP mutation (T1762/G1764)

## Discussion

It has been reported that the predominant HBV genotype in the Mediterranean area and the Middle East is genotype D [6]. Like other Iranian studies [7] all of our patients had genotype D. Unfortunately, patients with this genotype experience more severe disease courses and are less responsive to interferon therapy as compared to individuals with genotype A or B [8]. Additionally, the literature has established that genotype-D patients have higher HBV DNA levels [4]. The mutation from G to A in the PC region at nucleotide 1896 has been found in patients with HBeAg-negative chronic hepatitis [9]. This PC A1896 mutation creates a stop codon that prevents translation of the PC protein and abolishes the production of HBeAg. However, these patients continue to synthesize HBV DNA at sufficient levels to cause continual liver damage with progression to cirrhosis [10]. This type of mutation is detected in 20 to 95% of patients with HBeAg-negative chronic hepatitis B based on geographical variations [11]. The mutation is highly prevalent in Middle Eastern countries where genotype D is predominant. An Iranian study reported that 56% of patients with hepatitis B had a PC A1896 mutation [7]. The overall prevalence of this pattern of mutation in the Fars region for the current study was 31.8%, which is less than the abovementioned study. All the patients with PC A1896 mutation had simultaneous mutations at the BCP region. The other considerable point in our study was that all the patients had mutation from G to A at nucleotide 1896 (G1896A), and no other forms of mutations were observed in the PC region. This may be due to geographical differences and, secondly, to the age of our patients. The patients in other studies have generally been older with more chronic diseases; providing enough time for the PC region to replicate more and produce several patterns of PC mutations [12].

BCP mutation was detected in 17 (38.6%) of our patients. There were 3 patterns of BCP mutations in our patients. The most common form of BCP mutation was T1762/A1764 (25%), followed by A1762/A1764 and T1762/G1764, both with the same frequency (n = 3). Although previous studies have reported a higher prevalence of T1762/A1764 mutations in HBV genotype C patients [13], this pattern of mutation was more prevalent in our patients, all of whom had HBV genotype D infection. As previously mentioned in the methods section, we categorized our patients into 5 classes. Patients in Class 2, who had T1762/A1764 mutations, were older than the patients in other classes and had mildly elevated serum levels of AST and ALT.

This is compatible with other studies on older HBV patients [14]. However, Patients in class 3 (who had PC mutations at T1762/A1764) were the youngest among the 5 groups. It should be noted that due to the small number of patients in each class, a statistical comparison between classes was not possible. The other BCP mutations in our patients occurred in the A1762/A1764 region (Class 1) and at T1762/G1764 (Class 4). Patients with A1762/A1764 mutations (Class 1) had the highest serum levels of AST and ALT.The clinical and virologic significance of PC and BCP mutations are not fully understood. Some studies have shown an association between these mutations and more severe liver damage and hepatocellular carcinoma [5]. In the present study there were no significant differences by age, sex, and level of liver enzymes between patients with mutations and those without mutations. This finding may stem from the small number of patients in our study. Further studies with larger sample sizes are necessary to elucidate the exact role of these mutations in the clinical course of HBV infection.
